# An insight into two decades of Skilled Birth Attendants in Nepal

**DOI:** 10.3389/fgwh.2022.899010

**Published:** 2022-08-12

**Authors:** Shreyashi Aryal, Samata Nepal

**Affiliations:** ^1^Department of Obstetrics and Gynecology, Lumbini Medical College, Tansen, Nepal; ^2^Department of Community Medicine, Lumbini Medical College, Tansen, Nepal

**Keywords:** Skilled Birth Attendant (SBA), childbirth, Nepal, indicator, maternal mortality

## Introduction

Skilled Birth Attendants (SBA) in Nepal, comprising doctors, nurses, and midwives, have been providing skilled birth care and antenatal care since 2003. More than 7,000 trained SBAs have been working all over the country for almost two decades now, serving a population of almost 30 million (1). There has been a commendable decrease in maternal mortality ratio (MMR) by three quarters in these past decades, and this has been largely attributed to the coverage of institutional deliveries conducted by SBAs. Even though MMR has decreased, Nepal missed its MDG 5 target of MMR by 114. At the same time, paradoxically, maternal mortality has been increasing among institutional deliveries (2). As Nepal is in line to meet the SDG target, there is a need not just to increase institutional deliveries and consider that as an indicator for reduced maternal mortality, but also to assess the quality of care provided by SBAs.

## Current scenario

The government of Nepal allocated 2% as a share of GDP to healthcare for the Fiscal year 2019–2020 and this constitutes 5.4% for reproductive health and safe motherhood. A major share of 3.8% has been spent on training and workshops, which includes generous funds for the SBA training program (3). As a result, SBA training has been undergone robustly. Despite the production of SBAs and the continuity of care, the MMR curve has not descended significantly after 2017 with the COVID-19 pandemic expected to plateau the progression curve (4). There seems to be a need to discuss at this point whether SBA coverage is an indicator for decreased maternal mortality or whether it is a stale indicator (5).

To establish a direct correlation between decreased MMR and SBA attended deliveries, assessment of the quality of care is vital rather than the number of institutional deliveries. Maternal mortality is always preceded by complications. The measure of these complications in SBA vs. non-SBA attended deliveries might give a picture of the quality of care. Measuring center-specific complication rates may also help in analyzing preventable factors leading to maternal mortality, which constitutes 70% of all maternal deaths.

Nepal has 77.5% institutional deliveries with a target of 90% by 2030, among which only 79.3% of deliveries are conducted by SBAs (6). So, there are still many deliveries conducted at home either by unskilled birth attendants or Trained Birth Attendants (TBA). Underutilization of SBA for deliveries leads to a low volume of cases for trained SBAs. Currently, not all health centers in Nepal, including tertiary care centers, have SBA-attended deliveries. Many obstetric wards are run by nurses or Auxillary Nurse Midwives (ANM) without SBA training. So, measuring institutional delivery does not ascertain the quality of care.

SBA trainees in Nepal can either be doctors, staff nurses with a Proficiency Certificate Level after a 3-year training course, or ANM with an 18-month pre-diploma course. The 3-month training course consists of 27 core skills for managing normal deliveries and potential complications. The training materials for SBA are designed such that it requires basic proficiency in the English language as well. Basic educational criteria are critical to training efficiency, so reevaluation of the minimum criteria for enrollment in SBA training deems essential. After the training period, it has been seen that SBAs remain deficient in knowledge and clinical skill, so safe delivery cannot be ensured (7). It is also about time that the Nepalese reproductive health system starts focusing on the workforce with more elaborate training courses like the midwifery course or midwifery training for nurses.

## Discussion

There is no denying that the most critical intervention to reduce MMR is quality birth care or a minimum a timely referral system provided by SBAs. However, the competency of SBAs to be able to continue to do so for a number of years has to be assessed regularly. After training, SBAs go to remote areas and work as a lead in a low-resource setting. Some are posted in the government-established birthing centers, while some have started as a coordinator for their establishment. Recruitment of SBAs alone in those rural parts with limited infrastructures, inadequate equipment and drugs, the least professional support, and poor timely referral systems are some of the drawbacks to providing quality obstetric care (8). These can be the factors that cause pregnant women to bypass the lower-level health facilities. The WHO has a minimal recommendation for several deliveries to maintain competency, and this volume is not met in many of these settings. So, a refresher training for sufficient practice is necessary. The trainees are required to achieve an 80% standard to pass SBA training, and this standard must be maintained during follow-up as well. A regular follow and assessment of SBAs will help reinforce their skills. Supervision, accreditation, and continuing education are a must.

SBA training is based on having a wider public health care view and not only a focus on technical aspects of care. These aspects of SBAs have to be reinforced regularly. Antenatal coverage by SBA has increased but only 76% receive care as per the protocol (4). Obstetric violence is a contemporary issue that has been under scrutiny, so the quality of delivery care should be client directed. This should be incorporated in deliveries conducted by SBAs. Measurement of quality of care includes patient satisfaction, which is crucial in defining quality service provided by SBAs.

Like many low and middle-income Southeast Asian countries, Nepal has evolved with reproductive health gains from unattended home deliveries to deliveries attended by TBAs to Female Community Health Volunteers to SBAs. In a country like Nepal, where meeting MDG and SDG targets is a struggle, the role of SBAs is crucial in reducing maternal mortality. After moving to the federal government system, local governments can exercise the quality of the service provided at their municipality. Nepal has come a long way to increase access to reproductive healthcare for its women, but the key steps for the future would be to provide quality care through trained service providers, whose skills are assessed and reinforced regularly. With large investments of funds and workforce from the government, we need to check numbers, but quality check and patient satisfaction must be a priority.

## Author contributions

SA: conception and design and manusript draft preparartion. SN: draft preparation and critical revision. Both authors contributed to the article and approved the submitted version.

## Conflict of interest

The authors declare that the research was conducted in the absence of any commercial or financial relationships that could be construed as a potential conflict of interest.

## Publisher's note

All claims expressed in this article are solely those of the authors and do not necessarily represent those of their affiliated organizations, or those of the publisher, the editors and the reviewers. Any product that may be evaluated in this article, or claim that may be made by its manufacturer, is not guaranteed or endorsed by the publisher.
